# Comparison of two scales for evaluation of smile and dental attractiveness

**DOI:** 10.1590/2176-9451.20.2.042-048.oar

**Published:** 2015

**Authors:** Pedro Lima Emmerich Oliveira, Andrea Fonseca Jardim da Motta, Clarice Julia Guerra, José Nelson Mucha

**Affiliations:** 1 Masters student in Orthodontics, Universidade Federal Fluminense (UFF), School of Dentistry, Nova Friburgo, Rio de Janeiro, Brazil; 2 Associate professor, Universidade Federal Fluminense (UFF), School of Dentistry, Nova Friburgo, Rio de Janeiro, Brazil; 3 Postgraduate in Orthodontics, Universidade Federal Fluminense (UFF), School of Dentistry, Nova Friburgo, Rio de Janeiro, Brazil; 4 Full professor, Universidade Federal Fluminense (UFF), School of Dentistry, Nova Friburgo, Rio de Janeiro, Brazil

**Keywords:** Dental esthetics, Smile, Q-sort, Corrective Orthodontics, Visual analogue scale

## Abstract

**OBJECTIVE:**

To compare the visual analogue scale (VAS) and the simplified Q-sort method used to investigate the highest level of agreement among dentists, orthodontists and laypeople when assessing smile and dental attractiveness.

**MATERIAL AND METHODS:**

An album containing 258 photos of 86 individuals with their lips at rest, a slight and broad smile, was assessed by 25 dentists (general clinicians and various specialties), 23 orthodontists and 27 laypeople with regard to smile and dental attractiveness. To this end, both VAS and simplified Q-sort method were used. Agreements were calculated by intraclass correlation coefficient (ICC).

**RESULTS:**

For the single measurement between the VAS method and the simplified Q-sort method, all simplified Q-sort rates were higher in all groups. The simplified Q-sort method results ranged between 0.42 and 0.49 while those of the VAS method varied between 0.37 and 0.42. The simplified Q-sort method also presented higher mean measurement values (0.95 and 0.96) in comparison to VAS (0.94 and 0.95).

**CONCLUSIONS:**

Both scales may be considered reliable for evaluating smile and dental attractiveness; however, the simplified Q-Sort method presented slightly higher values than the VAS method.

## Abstract (pt)

**OBJETIVO:**

comparar a escala visual analógica (EVA) e o método Q-sort simplificado quanto à maior concordância nas avaliações entre cirurgiões-dentistas, ortodontistas e leigos em atratividade dentária e do sorriso.

**MÉTODOS:**

258 fotografias, provenientes de 86 indivíduos, fotografados com os lábios em repouso, sorriso leve e sorriso amplo, foram avaliadas quanto à atratividade dentária e do sorriso por meio da EVA e do Q-sort simplificado por 25 cirurgiões-dentistas (clínicos gerais e especialidades diversas), 23 Ortodontistas e 27 leigos. As concordâncias foram calculadas pelo Coeficiente de Correlação Intraclasse (ICC).

**RESULTADOS:**

para medida única entre a EVA e o método Q-sort simplificado, todas as taxas do Q-sort simplificado foram maiores em todos os grupos. O resultado do Q-sort simplificado variou entre 0,42 e 0,49, e da EVA entre 0,37 e 0,42. O Q-sort simplificado também apresentou valores de medida média superiores (0,95 e 0,96) em relação à EVA (0,94 e 0,95).

**CONCLUSÃO:**

pode-se considerar que ambas as escalas são confiáveis para avaliação da atratividade dentária e do sorriso; porém, o método Q-sort simplificado apresentou valores ligeiramente maiores que os da EVA.

## INTRODUCTION

One of the main objectives of orthodontic treatment is to improve the smile appearance.^1^ For this purpose, it is important to know the perception of orthodontists, dentists and mainly laypeople with regard to the ideal smile, in addition to bearing in mind that the definitive source of esthetic values must be related to the perceptions of the overall population, and not only to those of orthodontists and dentists.^2^^,^ 3

Thus, it is important to assess the perceptions of the overall population as well as professionals in Dentistry in order to determine some peculiarities common to all, or even reformulate some concepts about smiling, which would be more relevant.

Investigators have proposed different methods to assess esthetic concepts, each method with its advantages or limitations. The visual analogue scale (VAS) is one of the most popular and widely used method, probably because it is simple and inexpensive.^4^^-^ 9 It is used for esthetic evaluations of patient's profile,^9^ face,^8^ tooth positioning^4^^,^ 5 ^,^ 6 and post-treatment evaluations.^8^

The Q-sort method, developed by Stephenson in 1953,^10^ has been used in psychological and behavioral sciences,^11^ as well as to assess the esthetics of the smile^8^^,^ 12 ^,^ 13 and profile.^14^ In addition, there are methods based on scales of scores or ordinal categorization, such as the 10-point scale.^15^^-^ 18

Considering the availability of a high number of instruments of study, it is necessary to validate, compare and establish a gold standard for the methods of evaluating dentolabial attractiveness. The VAS method scores each object in an independent manner, while in the Q-sort method, the objects are evaluated in conjunction.^8^

Challenged by the question of which method should be used to assess the attractiveness of lip/tooth inter-relationship and smile, this article aimed to compare the scores assigned while assessing the attractiveness of photographs in an album from individuals with lips at rest, a slight and broad smile, by means of VAS and simplified Q-sort methods, and determine which types of evaluation presented the greatest reliability or less dispersive results.

## MATERIAL AND METHODS

The study was approved by the Institutional Review Board of the School of Dentistry of Universidade Federal Fluminense (UFF) under protocol #337193.

To conduct this study, a photograph album belonging to the Department of Orthodontics from Universidade Federal Fluminense (UFF) was used. The album comprised 258 color facial photographs of patients with lips at rest, a slight and broad smile. The photographs were obtained from 86 students enrolled in the undergraduate course in Dentistry at UFF, of whom 66 were females and 20 were males with an age-range from 19 to 30 years old.

In order to be included in the study sample, individuals should present complete permanent dentition from second molar to second molar, Angle Class I molar relationship, normal overjet and overbite, good facial profile tending to straight, no previous orthodontic treatment and could show teeth misalignment. From a total of 350 students enrolled in the dental school, 86 were selected and sex distribution is the real proportion of male and female students at that time.

Three photographs were obtained from each patient and standardized in the following manner: lips at rest, a slight and broad smile. A Minolta photographic camera with 100-mm macro lens was used. A Kodak 100 photographic film was used. The object-film distance was 1.0 m, with the head of each individual being positioned at the Frankfort plane parallel to the ground when the front view photograph was taken.

Images were digitized with an HP Scanjet G4050 scanner. Subsequently, Photoshop software (Adobe CS4, San Jose, California, USA) was used to diminish potential confounding factors. Cropping was done to limit the photographs to a restricted perioral area, excluding the nose, cheeks and chin. Potential rotations were corrected.^3^^,^ 18 ^-^ 24

To assess the photograph album in terms of attractiveness, a website was set up with the help of a programmer, and an online questionnaire was prepared. The participants were drawn from a list of dentists and orthodontists working in the city of Vitória, ES, Brazil. The laypeople had no specific knowledge about oral esthetics, or any education in sciences connected with the study of the face or art, such as plastic surgeons, estheticians and architects.^7^ They were postgraduate students attending public and private universities who were invited as volunteers.

Sample size was calculated on the basis of population estimations. The same parameters were used for the three groups (orthodontists, dentists and laypeople), and were as follows: 90% of confidence, error of 10% of the proportion in order to detect differences of 10% between groups. Thus, for a population of 140 orthodontists, we arrived at a sample of 23. For the population of 1643 dentists, the total number was 25, and as we had no population of laypeople, the sample was calculated without the factor of correction for the finite population, thereby arriving at a sample of 27.

**Figure 1 -:**
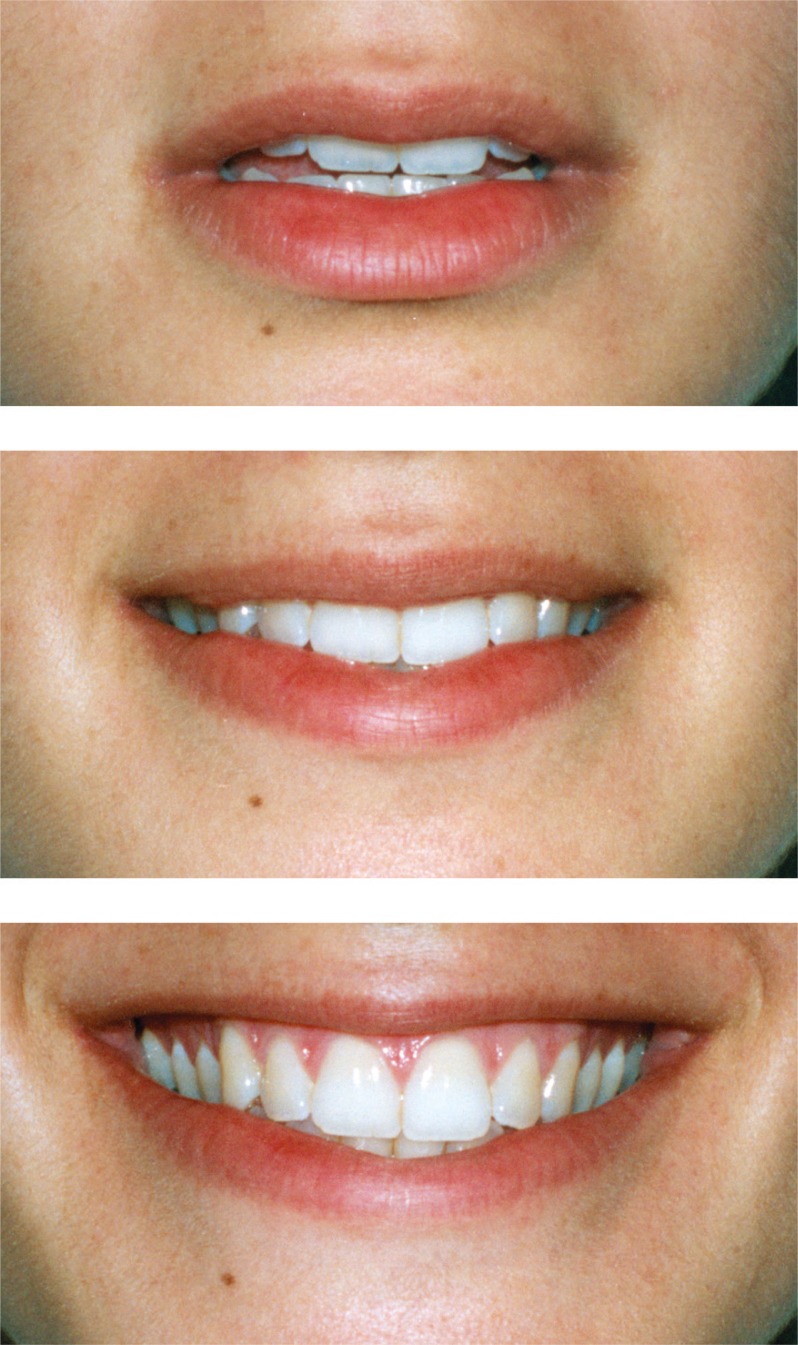
Illustration of 3 photos to be evaluated in the 3 categories.

Table 1 presents the distribution of the number and percentage of individuals in each group (dentists, laypeople and orthodontists). All individuals were instructed to take into consideration the set of three photos: at rest, with a slight and a broad smile (Fig 1).

**Table 1 -:** Descriptive analysis and percentage of individuals in each group.

		n	%
Individuals	Dentists	25	33.3
	Laypeople	27	36.0
	Orthodontists	23	30.7

**Figure 2 -:**
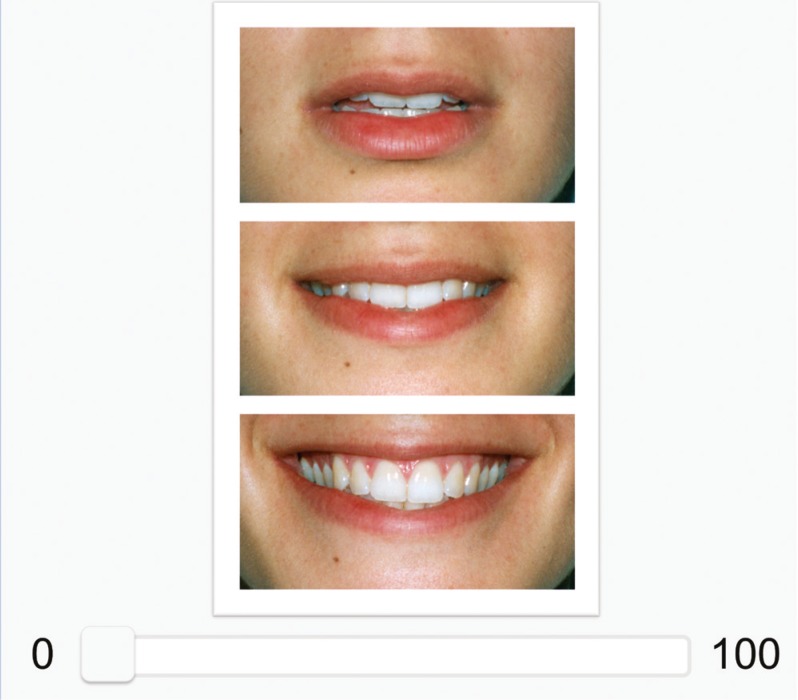
Illustration of the sliding bar (from 0 to 100) available on the website used to assess the value of attractiveness of the photos.

Each examiner assessed the photos twice, once by means of a visual analogue scale (VAS) and then again by means of the simplified Q-sort method. For the VAS method, a bar with a slider was developed on the website which the examiners used to position the point on the scale that represented the score in his/her judgment. Score "0" being the least imaginable level of attractiveness and "100" the most attractive level imaginable (Fig 2).

Stephenson^10^ proposed the Q-sort method with a ranked ordinal distribution into nine categories of a sample of 96 items evaluated, and Schabel et al^8^ applied the method to a sample reduced to 48 items. In this study, the simplified Q-sort method was used, in which the concept of ranked ordinal distribution was maintained with the arrangement changed from nine to five categories. The number of images evaluated was not pre-determined and could differ from 96 and 48.

For the simplified Q-sort method, the examiners received the following instructions adapted from the method performed by Schabel et al:^8^

1) Of the 86 images, select the 5 most and the 5 least attractive;
2) Of the remaining 76, select the 10 most attractive and the 10 least attractive;
3) The remaining 56 photos were automatically selected and considered as intermediate level of attractiveness.

The following scores were established: (0) 5 least attractive; (1) 10 least attractive; (2) 56 intermediate; (3) 10 most attractive; and (4) 5 most attractive.

After the photographs were assessed by the three groups of examiners in an independent manner, agreements between the VAS method and the simplified Q-sort method were calculated. To this end, the intraclass correlation coefficient (ICC)^25^ was used and evaluated as follows:

(a) Single measurement: one single measurement that evaluated the probability of an examiner generating the same scores for VAS and simplified Q-sort to be reproduced;

(b) Mean measurement: evaluated the probability of a group of examiners generating the same scores for VAS and simplified Q-sort to be reproduced;

Reproducibility, which measures the level of agreement between observations under the same circumstances, was assessed by means of analysis of variance (ANOVA) for the continuous scale (VAS), and the alternative non-parametric method to ANOVA, Kruskal-Wallis test, was used for the ordinal scale (Q-Sort), so as to verify the equality of means of scores in the two scales. The reproducibility assessed in this study was of the interobserver type; that is, by different evaluators, since the photos were not evaluated at two distinct time intervals.

The level of significance adopted in the test was 5% with a confidence interval of 95%. The software IBM SPSS Statistics version 19 performed the analyses.

## RESULTS

Means, standard deviation and maximum and minimum scores for VAS and simplified Q-sort methods are shown in Table 2. The means of VAS and simplified Q-sort scores were obtained by combining the results of the evaluators of each photo with its respective standard deviation. Score limits represent the minimum and maximum of a single evaluator.

**Table 2 -:** Descriptive analysis of scores stratified by categories of evaluators. Mean ± SD Minimum Maximum

		Mean ± SD	Minimum	Maximum
VAS method (0 - 100)	Dentists	45.34 ± 14.68	0	100
	Laypeople	37.18 ± 13.44	0	100
	Orthodontists	44.94 ± 11.78	0	100
Simplified Q-sort (0 - 4)	Dentist	2.00 ± 0.57	0	4
	Laypeople	2.00 ± 0.56	0	4
	Orthodontists	2.00 ± 0.58	0	4

When the VAS method was assessed, it was possible to observe that the group of laypeople was more critical towards the scores (37.18). In the general evaluation, the group attributing the highest scores were the dentists (45.34) followed by the orthodontists (44.94).

The evaluation between agreements is presented in Table 3. The ICC of the single measurement is an index used to demonstrate the confidence of the score in VAS and in the simplified Q-sort of one evaluator; whereas the ICC of the mean measurement determines the confidence by the mean of the score of a group of evaluators.

**Table 3 -:** Single measurement and mean measurement of the intraclass correlation coefficient (ICC) used to evaluate which of the two instruments (VAS and Q-sort) has the most robust scale or the one with the best reproducibility.

	Single measurement ICC		Mean measurement ICC	
	VAS	Simplified Q-sort	VAS	Simplified Q-sort
Dentists (n = 25)	0.42	0.47	0.95	0.96
Laypeople (n = 27)	0.37	0.42	0.94	0.95
Orthodontists (n = 23)	0.40	0.49	0.94	0.96

In the ICC (Table 3) of the single measurement for VAS, dentists (0.42) showed a higher level of agreement than orthodontists (0.40) followed by laypeople (0.37). In the simplified Q-sort, orthodontists showed the highest level of agreement (0.49), followed by dentists (0.47) and laypeople (0.42). In the ICC of the mean measurement evaluated by VAS, dentists (0.96) presented a higher level of agreement than orthodontists and laypeople (0.94). In the evaluation by Q-sort, dentists and orthodontists (0.96) obtained greater reproducibility than laypeople (0.95).

When observing only the single measurements between the VAS method and the simplified Q-sort method, all simplified Q-sort rates were higher for all individuals (dentists, laypeople and orthodontists). In this method, values ranged between 0.42 and 0.49, while VAS values ranged between 0.37 and 0.42. Therefore, results suggest that the simplified Q-sort method presents with more similar responses; that is, the method would be more reliable than VAS if evaluations were to be repeated.

The mean measurement, which is an index for a group of evaluators, ranged between 0.94 and 0.95 for VAS, and 0.95 and 0.96 for the simplified Q-sort method (Table 3), with equal variations in amplitudes, but of different magnitudes. Thus, the simplified Q-sort method would be considered slightly more reliable than VAS, if new measurements were to be made.

ANOVA and Kruskal-Wallis tests (Table 4) demonstrated that there were no statistically significant differences between the means of scores awarded by the evaluators in the three groups, which demonstrates good interobserver reproducibility. However, p values of the Q-Sort method were higher; thus, its reproducibility is considered better when compared to VAS.

**Table 4 -:** Interobserver reproducibility of scores.

	P value	
	VAS¹	Q-sort ²
Dentists (n = 25)	0.145	0.888
Laypeople (n = 27)	0.201	0.902
Orthodontists (n = 23)	0.120	0.805

¹ ANOVA.

² Kruska-Wallis.

## DISCUSSION

Mean values were lower than 50 for all groups (Table 2). Howells and Shaw^26^ and Schabel et al^8 ^found mean VAS values near 50, which is in the center of the scale. This difference may be justified by the characteristics of the sample. The current sample did not include individuals who had undergone orthodontic treatment, unlike the sample from Schabel et al.^8^ However, the values obtained corroborate the affirmative finding that evaluators tend to attribute scores that remain distant from the extremity of the scale.^8^

Based on the mean values obtained in VAS, we observed that dentists and orthodontists attributed higher scores in comparison to laypeople. Zange et al^4^ also found laypeople to be more critical than orthodontists when VAS was used. Guo et al^23^ found that oral and maxillofacial surgeons were stricter than laypeople when evaluating gingival smile; and Elham et al^27^ found that laypeople were less demanding than dentists and orthodontists. These differences in mean values may be justified by two reasons: firstly, the differences in study designs, since digitally modified sequential images were used in those studies; secondly, laypeople may have evaluated facial characteristics other than smile and dental attractiveness, even though images had been cropped to a specific and restricted area.

Although laypeople have a lower average score that indicates higher criticism in assessment, they also have less agreement, particularly in the single ICC measurement. Even with a small difference, orthodontists and dentists were more consistent.

By means of Q-sort, it was not possible for the mean value of the evaluation to differ from four.^8^ In this study, due to the simplification and modification of the scale, scores between 2.05 and 1.83 were attributed because the subjects were not normally distributed, as they would have been in the original method.

The limited aspect of the study is related to the absence of socioeconomic inclusion or exclusion criteria and the selection of the sample of laypeople with no randomization. However, these factors do not invalidate the results, especially in the selection of laypeople, since the ICC had values similar to orthodontists and dentists.

The purpose of the ICC was to evaluate whether the scales presented confidence for studies with regard to perception of dentolabial esthetics, in addition to showing which scale would be superior to be used in future studies. The VAS method has been used in other investigations and is a tool of proven scientific validity. The results of the mean measurements were 0.94 and 0.95 in a maximum coefficient of one; therefore, the reliability of the scale was confirmed. Schabel et al^8^ proved the reliability of the Q-sort method and also found higher agreement than the VAS method when it was used in a single ICC measurement and in the mean measurement. Moreover, the values obtained for the mean measurements in clinical photographs were the same as those found in the present study (0.95 and 0.96). The simplified Q-sort method (0.95 and 0.96) of the present study also proved reliable and presented a slightly higher ICC for both single and mean measurements than those found for VAS.

When assessing agreement in each professional group, as shown in Table 3, dentists presented a higher level of agreement in the ICC of the single measurement of VAS (0.42), mean measurement of VAS (0.95) and simplified Q-sort (0.96). Orthodontists obtained higher scores of the ICC single (0.49) and mean (0.96) measurements in the simplified Q-sort. Laypeople presented a lower level of agreement in comparison to the other groups in all methods; nevertheless, with an excellent ICC score for the mean measurement (0.94 and 0.95). Although in a different esthetic and statistical context, Gehrke et al^28^ found a higher level of agreement between orthodontists and a lower level of agreement between laypeople. In an experiment with ICC for profile evaluation, Sloss et al^29^ also found strong agreement between residents in Orthodontics and laypeople.

The force of the mean measurement values found for ICC may be justified by the number of evaluators (n = 75),^8 ^since the ICC tends to increase as more evaluators are added.^26^ Single measurement scores are lower than those of the mean measurement because they refer to a pair of data collected, while the mean measurement refers to the entire group.

Although the Q-sort method applied in this study was simplified and modified, it still presented similarity to the original: a ranking mechanism alternating with pre-established scores. The ranking mechanism represents the greatest distinction between VAS and Q-sort, and this is probably the reason why minor differences in confidence were found between scales. The results and the difference in values ​​found do not invalidate any scales, but corroborate the studies using them. Both can be displayed or interpreted for clinical practice. In spite of being executed in a different manner in comparison to VAS, the Q-sort or simplified Q-sort method is understandable and uncomplicated. Additionally, because they present a slightly higher level of agreement between evaluators, they could be considered the first choice as a method of scientific evaluation with regard to dentofacial attractiveness.

## CONCLUSIONS

When dentists, orthodontists and laypeople evaluated the attractiveness of photographs with lips at rest, slight and broad smile by means of VAS and the simplified Q-sort method, both scales proved to be reliable. However, the simplified Q-sort method presented a slightly higher level of interobserver reliability in comparison to VAS, and should, therefore, be preferred as a method for evaluation of smile and dental attractiveness.
